# A preliminary, prospective study of peripheral neuropathy and cognitive function in patients with breast cancer during taxane therapy

**DOI:** 10.1371/journal.pone.0275648

**Published:** 2022-10-07

**Authors:** Eiman Y. Ibrahim, Saira Munshani, Ilaria Domenicano, Rozalyn Rodwin, Richard J. Nowak, Lajos Pusztai, Maryam Lustberg, Barbara E. Ehrlich

**Affiliations:** 1 Department of Pharmacology, Yale University, New Haven, CT, United States of America; 2 Department of Biostatistics, Yale School of Public Health, New Haven, CT, United States of America; 3 Department of Pediatrics (Hematology/Oncology), Yale School of Medicine, New Haven, CT, United States of America; 4 Department of Neurology, Yale School of Medicine, New Haven, CT, United States of America; 5 Department of Medicine (Medical Oncology), Yale School of Medicine, New Haven, CT, United States of America; BG-Universitatsklinikum Bergmannsheil, Ruhr-Universitat Bochum, GERMANY

## Abstract

Dramatic improvements in cancer survival have occurred in the last decade, but the quality of life for many survivors is compromised due to severe, long-lasting, and often irreversible side effects of chemotherapy. The neurological side effects, chemotherapy induced peripheral neuropathy (CIPN) and cancer related/induced cognitive impairment (CRCI/CICI), are under-recognized and can occur after chemotherapy, immunotherapy, or radiation. The cellular mechanisms underlying these neurological side effects are poorly understood and there are no effective treatments or preventions, other than reduction or termination of cancer therapy. In our preliminary prospective, non-interventional study to examine the side effects of chemotherapy in patients with breast cancer (NCT03872141), patients with breast cancer who received standard of care single agent weekly taxane-based chemotherapy were assessed at baseline, midpoint, and end of treatment for neurological and cognitive changes and for blood levels of potential protein biomarkers (n = 13). CIPN and CRCI both showed an increase in severity with accumulating taxane and these changes were compared to protein alternations over the course of treatment. Using peripheral blood collected from patients (n = 10) during chemotherapy and tested with an antibody array curated by the MD Anderson RPPA Core), we found that 19 proteins were increased, and 12 proteins decreased over 12 weeks of treatment. Among those downregulate were proteins known to be critical for neuronal viability and function including GRB2 (growth factor receptor-bound protein 2) and NCS1 (neuronal calcium sensor 1). Concurrently, proteins associated with apoptosis, including BAK1 (Bcl-1 homologous antagonist/killer), were upregulated. These results support the proposal that CIPN and CRCI increase with increasing taxane exposure, and identified several proteins that are altered with taxane exposure that could be implicated in their pathogenesis. In conclusion, our study provides evidence for progressive neurological changes and the rationale to investigate the molecular basis for these changes with the goal of target identification for mitigation of these neurological side effects.

## Introduction

Breast cancer is the most common cancer in women worldwide and is still considered the second leading cause of cancer death in developed countries despite the advances made in the field of cancer care over the last two decades [1,2]. There has been substantial improvement in survival of patients with breast cancer due to the development and use of novel endocrine therapies, chemotherapies, and targeted immunotherapies. Although some single agent therapies are proving to be effective in specific tumor types, only a limited number of patients have access to and are benefiting from these therapies. Until the effectiveness of targeted therapy is expanded, chemotherapy will remain essential for patients with breast cancer. Overall, breast cancer continues to be considered a challenging disease and prognosis varies widely among patients [3,4].

Taxanes such as paclitaxel and docetaxel are among the most effective agents and various combinations have been tested for optimizing results [5]. These compounds act by promoting polymerization of tubulin into highly stable intracellular microtubules, a process that disrupts mitosis and normal cell division, and eventually leads to cell death [6]. Such agents have significantly improved survival rates of patients and may reduce the risk of toxicities from other common chemotherapies [7]. Nevertheless, these agents have been associated with specific neurological adverse events. Chemotherapy induced peripheral neuropathy (CIPN) is among the most common of these events with prevalence up to 87% and it poses a challenge for both patients and health care providers [8]. More recently, taxane-induced cognitive impairment was found to be common in the domains of attention, executive function, and depression especially six months or more after a full course of treatment [9].

Some of these neurological impairments are irreversible and represent a major challenge for physicians because it is difficult to distinguish or predict the susceptibility of individual patients to develop neurological symptoms. Also, there are no early signs or indications warranting a reduction in the dosage to alleviate the severity of any impending adverse responses. Once pain and sensory abnormalities, as well as cognitive defects occur, they often persist for months or even years after the cessation of chemotherapy due to the inability of nervous tissues to regenerate. In sum, patients may be cured from cancer using taxanes but may suffer for decades from debilitating neuropathy induced by these cancer therapies.

The cellular processes that have been proposed to explain how taxanes cause CIPN include induction of neuroinflammation and alteration of cell signaling, mechanisms which can lead to alterations in peripheral nerve excitability [8]. It has also been found that taxanes can cross the blood-brain barrier and accumulate in the central nervous system (CNS) [10]. Recent studies focus on improving delivery of taxanes into the brain to treat brain metastases and cancers [11,12] underscoring the need to better understand taxane toxicity in the CNS. We previously outlined a mechanism for paclitaxel-induced CIPN [13,14] and chemotherapy related cognitive impairment (CRCI) [15] and found agents that could prevent neuropathy in mouse models [15,16]. We found that paclitaxel binds to neuronal calcium sensor 1 (NCS1) [17] which is a key regulator of neuronal signaling [18], in part through binding to the inositol trisphosphate receptor (InsP3R) [13]. Briefly, when paclitaxel binds to NCS1 it enhances NCS1/InsP3R-dependent calcium release from the endoplasmic reticulum which leads to elevated intracellular calcium. This increased cellular calcium activates the pro-apoptotic protease calpain, cleaving proteins necessary for neuronal function. The current research provides compelling evidence that both peripheral neuropathy and cognitive impairment have mechanistic similarities leading to the hypothesis that it will be possible to develop targeted treatments to alleviate side effects of taxane treatment.

The purpose of the current prospective pilot study (NCT03872141) was to assess neurological and cognitive changes in 13 patients with breast cancer who received standard of care single agent weekly paclitaxel or docetaxel chemotherapy and to correlate protein biomarker changes in peripheral leukocytes with development of CIPN and CRCI. Although this study has a small sample size, it provides a comprehensive assessment of nerve conduction measurements, neurocognitive assessment, patient reported neuropathy measures, and surrogate biomarkers longitudinally during 12 weeks of taxane therapy.

## Materials and methods

### Study design and patient recruitment

**Fig 1:**
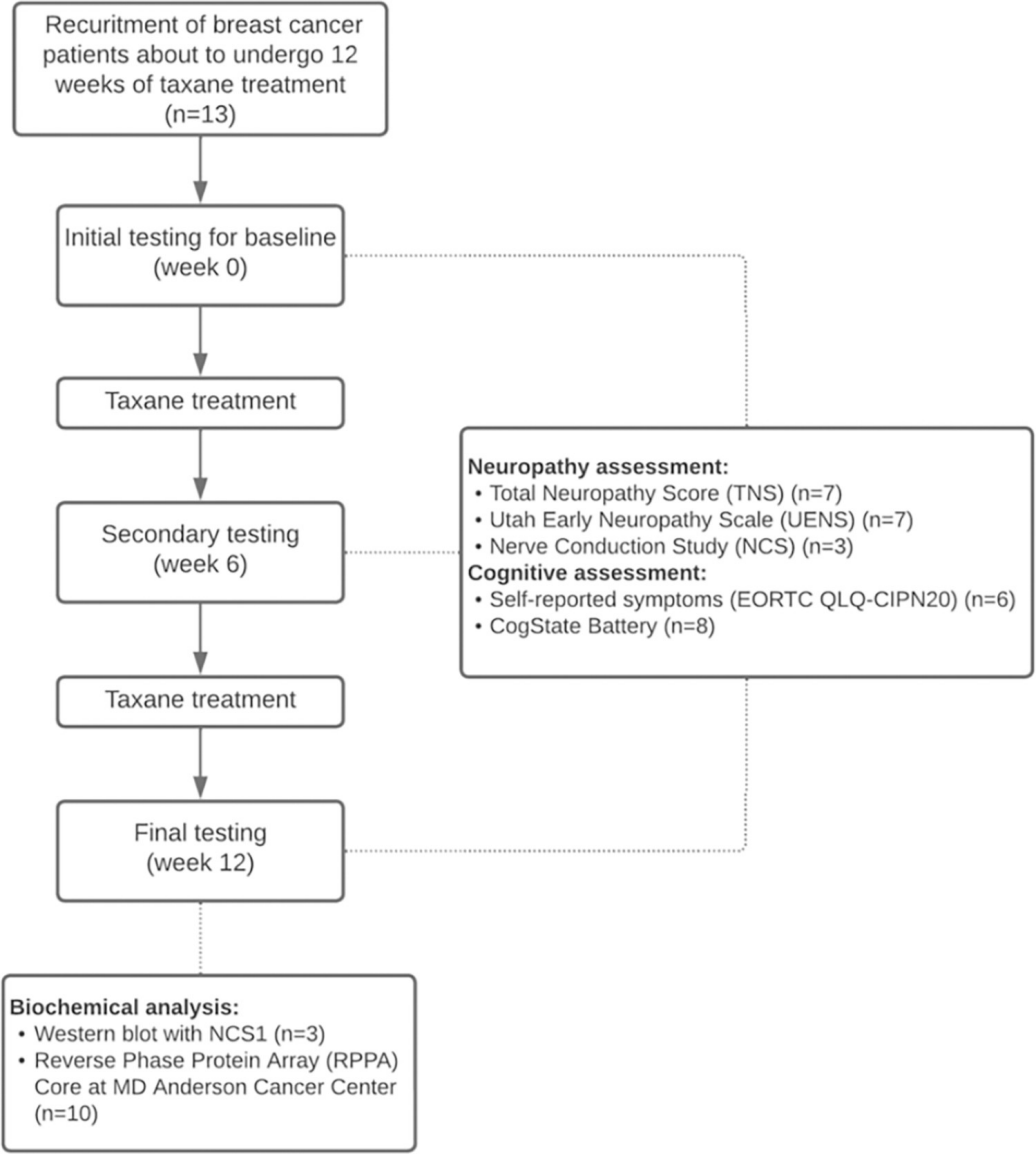
Schematic of study assessments.

Newly diagnosed stage I-III patients with breast cancer who were starting standard of care adjuvant or neoadjuvant chemotherapy with weekly paclitaxel or docetaxel were identified by their treating oncologist and referred for clinical and neurophysiological assessments. Demographic data, medical history, including treatment and previous diagnosis of any comorbidities were collected. Studies were approved by the Yale Cancer Center Human Research Ethics Committee (NCT03872141) and written informed consent was obtained in accordance with the Declaration of Helsinki. This study was designed to evaluate neurological and cognitive function to determine the onset and severity of CIPN and CRCI. Using clinical testing, self-reported data, and biochemical results (**Fig 1**), we aimed to understand the effects of neuropathy on a systemic and cellular level. We performed assessments over the course of the treatment at three time points (baseline, 6-week and 12 week). When appropriate, patients were used as their own control.

### Inclusion criteria

1. Women ages 18 to 70 years old
2. Histologically confirmed clinically stage I-III breast cancer
3. Receiving weekly paclitaxel (80 mg/m^2^) chemotherapy as part of their routine care.

### Exclusion criteria

1. Patients with known prior history of neuropathy or symptoms of numbness or peripheral pain, or prior neurotoxic chemotherapy
2. Patients who suffer from diabetes mellitus or current symptoms of numbness and neuropathic pain
3. Treatment for bipolar disease
4. Treatment or concomitant use of common medications used to treat neuropathic pain, such as amitriptyline, gabapentin, pregabalin, duloxetine, for any indication
5. Limited English that would preclude understanding and completion of the study questionnaires
6. Pregnancy
7. Life expectancy <12 weeks
8. Participation in other research studies either concurrent with or within 30 days prior to participation in this study.

Patient demographics, including age, race, smoking history, BMI, chemotherapy agent received, and setting the agent was received in, are summarized in **Table 1**.

**Table 1:** Breast cancer patient characteristics (n = 13).

VARIABLE	VALUE (n = 13)
**AGE**	
All	52 ± 11.8
**BMI**	
**All**	29.2 ± 7.4
**RACE**	N (%)
White	10 (77)
Hispanic	2 (15)
Black	1 (8)
**BREAST CANCER**	
Stage I	3 (23)
Stage II	7 (54)
Stage III	2 (15)
**AGENT RECIEVED**	
DDAC then Taxol x12	4 (31)
Taxol 80x12 then DDAC	4 (31)
OPAC then Taxol 80x12	2 (15)
Docetaxel + Trastuzmumab + pertuzumab	1 (8)
Taxol x12 then radiation	1 (8)
**SETTING**	
Neoadjuvant	9 (69)
Adjuvant	4 (31)
**MENOPAUSAL**	
Positive	6 (46)
**HISTORY OF SMOKING**	
Positive	7 (54)

### Assessment of neurotoxicity and CIPN

Assessment of neurotoxicity and CIPN were split into two categories: patient reported outcomes (subjective) and clinical evaluation (objective). These are both detailed below and were obtained at baseline, 6 weeks, and 12 weeks after initiation of taxane-based chemotherapy.

- Patient reported outcomes
  *(EORTC) QLQ-CIPN20*: The European Organization of Research and Treatment of Cancer (EORTC) QLQ-CIPN20 questionnaire [19] is a patient self-report questionnaire with a subsection of questions that addresses specific daily function (i.e., trouble opening a jar or holding a pen). This questionnaire appears to be more sensitive in identifying sensory symptoms, which are the more debilitating features of CIPN [20] (sensory scale range 1–36, motor scale range 1–32). The raw scores were linearly converted to a 0–100 scale based on the raw scale values.
- Physician reported outcomes.
  All clinical evaluations were completed by Dr. R. Nowak in the Yale Neurology Suite.
  *Total Neuropathy Score (TNS) [21]*: The TNS is a composite score incorporating subjective symptom reporting, a formal neurologic examination, qualitative vibration sensory testing, and electrodiagnostic testing. TNS testing has been shown to be reliable and superior to other tools in assessing the severity and changes in CIPN. Higher scores indicate greater severity of neuropathy (range 0–24).
  *The Utah Early Neuropathy Scale [22]:* This assessment is a physical examination to detect early sensory predominant polyneuropathy through pin prick sensitivity, vibration and position sensation, motor examination, and reflex testing. Higher scores indicate greater severity of neuropathy (range 0–42).
  *Nerve Conduction Study (NCS)* [23]: The nerve conduction study measures the response size and speed of electrical impulses applied through the skin at different points to assess overall nerve health and function. The NCS allows us to more directly measure the effects of chemotherapy on peripheral nerves, specifically large-fiber nerves. amplitude. The peroneal motor nerve and sural sensory nerve were evaluated in this study. The peroneal motor study was conducted to the extensor digitorum brevis (EDB) muscle. The nerve was stimulated approximately 7 cm proximal to the active recording electrode and also stimulated at the fibular head. For the sural sensory study, the active electrode was placed posteroinferior to the lateral malleolus. The stimulus was given at 14 cm proximal to the active recording electrode, slightly lateral to the midline of the posterior calf. Both studies included a reference electrode and ground electrode per standard. The skin temperature was monitored during the study and was maintained between 30°C−33°C. Analysis was limited to the following parameters: sensory conduction velocity (Sensory CV), sensory nerve action potential amplitude (SNAP AMP), Motor Distal Latency, motor conduction velocity (Motor CV), and compound muscle action potential amplitude (CMAP AMP). All reference values were based on the American Association of Neuromuscular & Electrodiagnostic Medicine (AANEM) consensus guidelines and standards. The Dantec® Keypoint® Focus EMG / NCS / EP System was the instrument and software used for the study (Natus Medical Incorporated, USA).

### Assessment of cognitive function

The CogState platform (https://www.cogstate.com/) was used to assess cognitive function based on simple tasks that test attention, learning, and memory [24]. All CogState tasks were performed at baseline, 6 weeks, and 12 weeks from the start of taxane therapy. CogState has identified a set of measures that are optimal for the detection of cognitive change in clinical trials at both the group and individual level. The tests were performed at the infusion unit at Smilow Cancer Center. The battery of cognitive tasks was selected in consultation with CogState associates and were chosen because they reflect different cognitive functions, relevant to different brain regions. The selected CogState tests included the “Detection,” “Identification”, “One Card Learning,” “One Back Speed,” and “One Back Accuracy” tests.

### Assessment of biological markers

#### Western Blot for neuronal calcium sensor 1 (NCS1)

Blood samples were collected in heparinized tubes before the first paclitaxel/docetaxel treatment (baseline) and at 6 weeks and 12 weeks during treatment. White blood cells were isolated, frozen and stored at -80C. White blood cells were selected as proxy for neuronal NCS1 given the ease of collection and correlation between NCS1 levels in two tissues from the same subject (neuronal and breast) from the GTex portal data base. The comparison between breast tissue and neuron expression was chosen because the database contained sufficient numbers of patients with NCS1 expression in these two tissues. We hypothesize this correlation would apply to other tissues including white blood cells (https://gtexportal.org/home, **S1 Fig**).

Using standard western blot methods, NCS1 and beta-actin (used as a loading control) levels in white blood cells were quantified and compared [25]. Briefly, as previous reported [15,16], blood samples were thawed in RIPA buffer (NaCl (150 mM), Nonidet P-40 (1%), deoxycholate (DOC, 0.5%), sodium dodecyl sulfate (SDS, 0.1%), Tris (50 mM, pH 7.4)) containing protease inhibitor, phenylmethylsulphonyl fluoride (PMSF), and sodium orthovanadate (Santa Cruz) and then spun down at 13000 rpm, 4°C to remove cell debris. Protein concentration was quantified using Pierce BCA protein assay kit (ThermoFisher Scientific) according to the manufacturer’s instruction. Western blots were performed using the NuPAGE system (ThermoFisher Scientific) and PVDF membrane with the Biorad wet transfer system (Bio-Rad Laboratories). Approximately 20 μg total protein was loaded into each lane. Original blots show responses to staining by antibodies to NCS1, GAPDH, neurofilament light chain, and GAPDH antibodies. Results from neurofilament light chain, and GAPDH were not included in the analysis. Antibodies used were anti-NCS1 (Santa Cruz, FL-190) and anti-beta-actin (Cell Signaling 8H10D10).

#### Reverse Protein Proteomics Array (RPPA)

White blood cell aliquots were collected from the patients at baseline, 6 weeks, and 12 weeks during treatment. These samples were sent to the Protein Array and Analysis Core of MD Anderson Cancer Center, Houston, TX for assessment. The RPPA core facility at MD Anderson Cancer Center used a set of 484 predetermined antibodies for each array, and the results were quantified by a set of MD Anderson algorithms. The raw data for protein expression levels was analyzed using standard web-based protocols [26].

### Statistical analysis details

Patient characteristics were evaluated using simple descriptive statistics. For patients whose NCS measures were obtained, we computed the changes from baseline to 12 weeks. Sensory CV, SNAP AMP, Motor Distal Latency, Motor CV, and CMAP AMP were the variables used to assess the effects of chemotherapy on peripheral nerves. Linearly transformed scores were reported for the EORTC at baseline, 6 weeks, and 12 weeks for those patients who completed the questionnaire at all the three time points of the study. The average relative changes of CogState Assessments over the course of 12 weeks were computed for “Identification”, “One Card Learning,” and “One Back Accuracy” when data were available. Lastly, NCS1 Levels were computed at baseline, week 6, and week 12 as the ratio between NCS1 and β-actin for those patients with no missing values. Boxplot and summary statistics were included for NCS, EORTC, CogState, and NCS1 levels at each time point. Dues to the limited sample size imputation was not performed for missing values. Relevant statistical parameters are reported in figure legends and at the end of this paper (**S1 Table**).

## Results

### Patient cohort and study set-up

Thirteen patients with breast cancer over the course of a 12-week docetaxel or paclitaxel treatment were monitored and tested (**Fig 1**). Patient characteristics are reported in **Table 1.** All patients who showed up for repeat testing (baseline, 6 weeks and 12 weeks) were evaluated. Not all tests were done on every patient. Blood collection, CogState tests, and self-reporting questionnaires were performed in the infusion center, a controlled clinical setting. Clinical evaluation of CIPN was done in the Yale Neurology Suite.

### Chemotherapy-induced peripheral neuropathy

**Fig 2:**
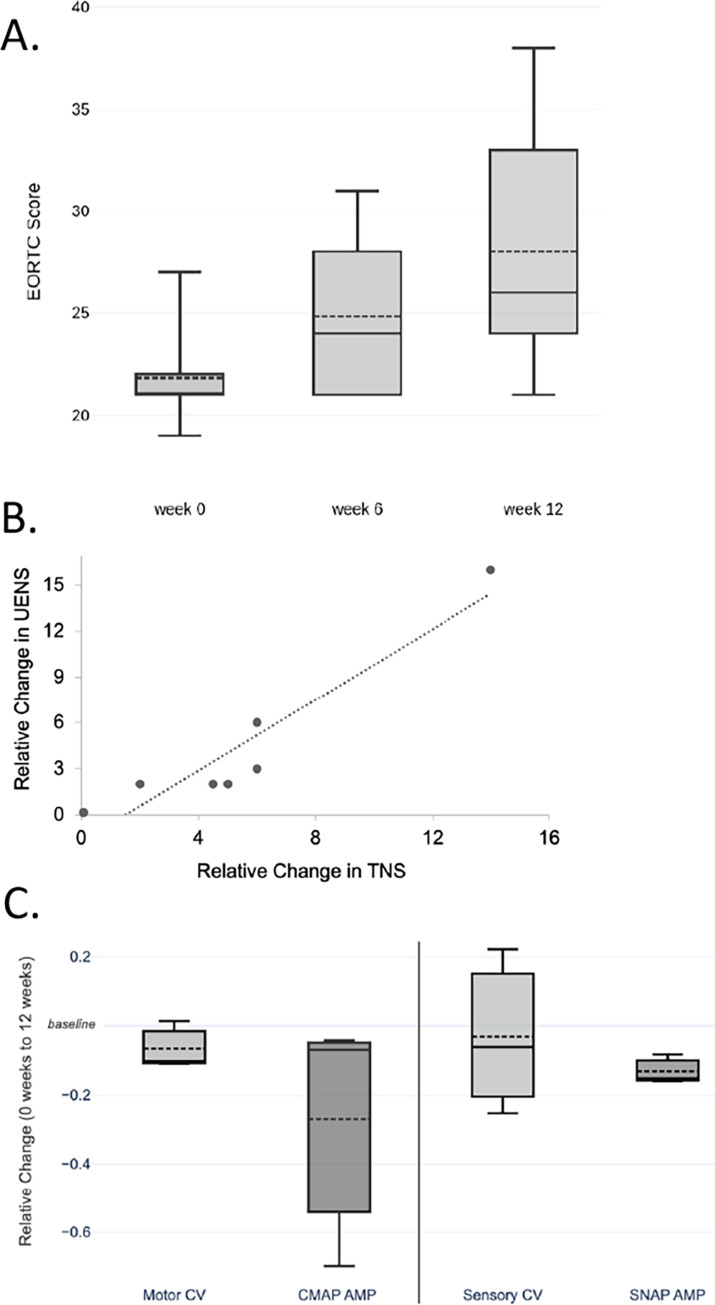
Measurements of CIPN. **A**. The EORTC-QLQ-CIPN20 questionnaire score over 12 weeks of treatment. This is a self-reported indicator of CIPN. Higher values indicate more severe CIPN symptoms (n = 6 at each time point). **B.** Changes in Total Neuropathy and Utah Early Neuropathy Scores over the 12-weeks of treatment. For both tests, higher scores (more positive values) indicate higher severity of symptoms. Each point represents an individual subject (n = 7 at each time point). Note that the two measures of CIPN are positively correlated. **C.** Nerve conduction study (NCS) results (n = 3 at each time point) showing change in measurements from baseline for peroneal motor nerve and sural sensory nerve. CMAP = compound muscle action potentials (uV), CV = conduction velocity (m/s), SNAP = sensory nerve action potentials (uV), AMP = amplitude (uV). In panels A and C, dark line indicates median of data, the upper and lower bounds of the boxes are quartile 1 (Q1) and quartile 3 (Q3) respectively, and dashed lines extend to and lower values showing the 95% confidence level (CL).

Seven patients self-reported their status as assessed by the EORTC-QLQ-CIPN20, and 6 of these 7 patients reported peripheral neuropathy symptoms, such as tingling and numbness in the hands and feet (**Fig 2A**). Higher values are indicative of more severe CIPN symptoms. The scale was linearly transformed to a 0–100 scale, based on the highest and lowest possible EORTC scores, which is consistent with previously reported studies [27]. The EORTC-QLQ-CIPN20 score increased on average from 29 +/- 3.3 to 37 +/- 7.7 (p = 0.028, paired t-test) over the course of 12 weeks (**S1 Table**). The increase in the EORTC-QLQ-CIPN20 score over 12 weeks indicates that during chemotherapy patients noticed CIPN related symptoms affecting their daily function. Although this questionnaire is subjective and self-reported, the results align with prior reports [28] and the present clinical findings (see next section).

The Total Neuropathy Score (TNS) and Utah Early Neuropathy Score (UENS) values for seven patients from the cohort were collected and subsequently analyzed and correlated (**Fig 2B**). Both the TNS and UENS scores indicated deterioration of neuronal function for all patients tested (n = 7). The change in scores over the 12 weeks for TNS and UENS were similar (relative change for TNS = 5.3 +/- 5.1 and relative change for UENS = 4.4 +/- 4.7), and the positive correlation in these tests provide validation of CIPN in the patient cohort.

Nerve Conduction Study (NCS) data at 0 weeks and at 12 weeks were used to interpret changes over the course of treatment (n = 3). Motor conduction velocity, CMAP amplitude, sensory conduction velocity, and SNAP amplitude were considered for the motor peroneal and sensory sural nerves (**Fig 2C**). The average relative changes during the study were -0.031 +/- 0.19 for sensory CV, -0.132 +/-0.03 for SNAP AMP, -0.049 +/- 0.11 for motor distal latency, -0.067 +/- .05 for motor CV, and -0.269 +/- 0.30 for CMAP AMP (**S1 Table**).

### Chemotherapy-induced cognitive impairment

**Fig 3:**
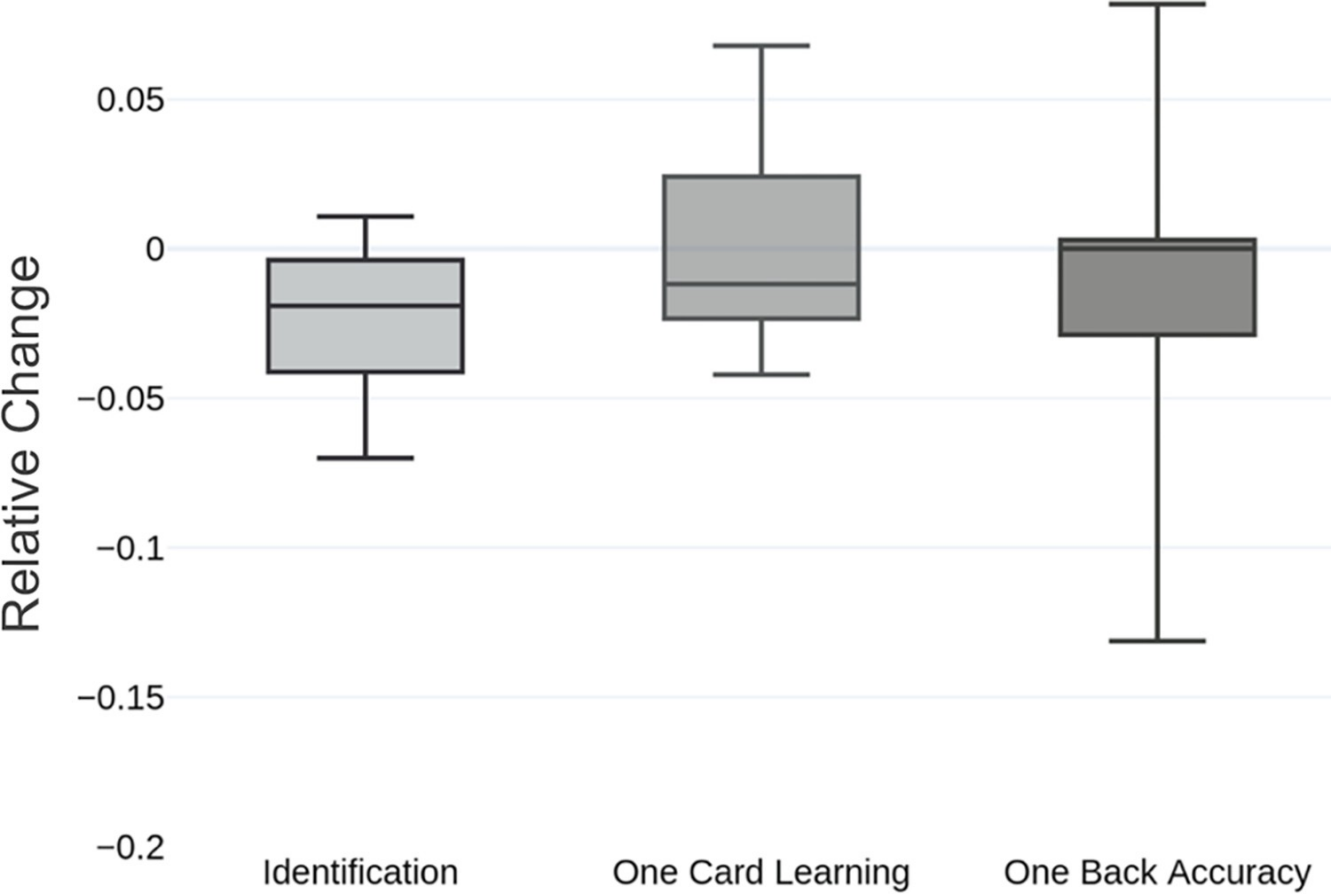
CogState test scores over 12 weeks of treatment. Average relative changes of CogState test scores over course of 12-week treatment. The decreased average relative change indicates that there was a decrease in test performance across the tested cohort (n = 8 at each time point).

The results of the CogState tests varied among the 12 patients tested, but several tests showed a decrease across the cohort (**Fig 3**). The “Identification Test,” which measures speed of performance and analyzes attention, the “One Card Learning Test,” which measures accuracy of performance and analyzes visual learning, and the “One Back Accuracy Test,” which measures speed and accuracy of performance and analyzes working memory, all had an overall decrease in patient performance across the treatment period (relative changes were -0.023 +/- .02, -0.0122 +/- 0.01, and -0.0127 +/- .05, respectively). The “Detection Test”, which measures speed of performance and analyzes psychomotor function and “One Back Speed,” which measures speed of performance and analyzes working memory, did not show consistent changes among the cohort.

### Molecular alterations/biomarkers in the blood of taxane-treated cancer patients

**Fig 4:**
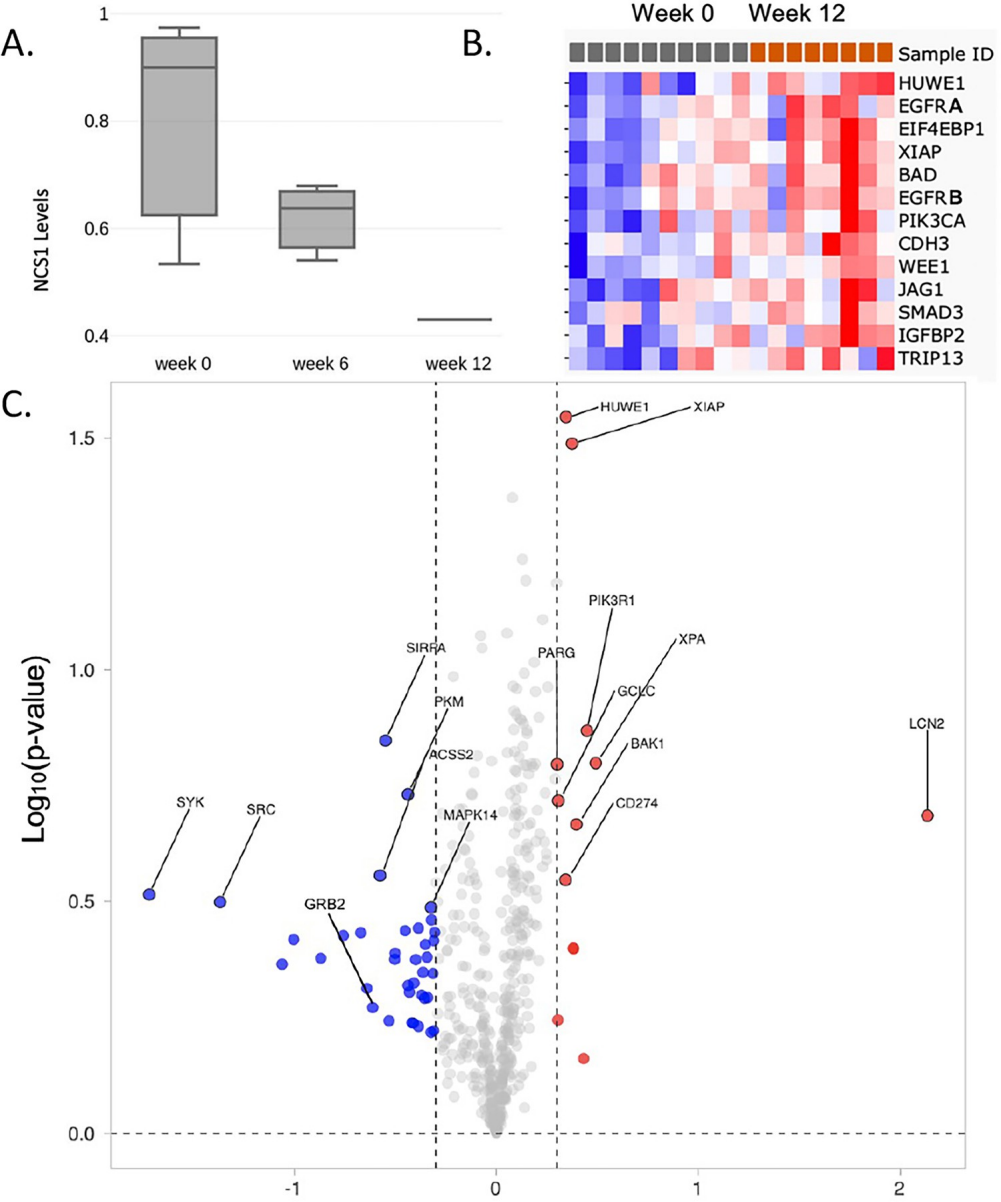
Measurement of protein expression. **A.** NCS1 levels over the course of 12 weeks of treatment. Western blot assessment of NCS1, using beta-actin as a loading control, in samples from patient white blood cells (n = 3 for weeks 0 and 6). Only one blood cell sample was available for assessment at week 12. The individual values for NCS1 (using beta-actin as a loading control) are: Baseline—0.97, 0.53, 0.90; week 6–0.67, 0.54, 0.63; week 12–0.43 **B.** Heatmap with proteins which had a 35% or greater increase in expression from the start of treatment (week 0) to completion (week 12). Sample ID refers to individual patients (n = 10). In the top line, gray boxes are baseline values, orange boxes are values after 12 weeks of treatment. Refer to Table 2 and panel C for further information. The progression from blue to red indicates that the protein levels go up relative to their baseline values. EGFR is listed twice (EGFRA and EGFRB) because two different EGFR related antibodies were included in the RPPA core tests. **C.** Volcano plot showing changes in protein levels as assessed by the MD Anderson Cancer Center RPPA Core using patient white blood cell samples (n = 10). Blue dots represent proteins which had an overall decrease, and red dots represent proteins which had an overall increase. Normalized protein levels were mean centered and a threshold of log2 (Fold Change) of (-.35,.35) was used (dashed line). No significance threshold is depicted in the graph, but the further up a protein is on the y-axis, the more significant of a change. P-values were not adjusted.

To determine whether the NCS1-dependent molecular pathway of CIPN identified in preclinical models [13,14,29] was reflected in human subjects, NCS1 protein levels were assessed by western blot using patient white blood cell samples (n = 3). The observed changes indicated that there was a decline in NCS1 levels from baseline to 6 weeks to 12 weeks (0.8 +/- 0.19, 0.61 +/- 0.05, 0.43, respectively). (**Fig 4A**). In this report, NCS1 levels in peripheral blood are included as a proxy for neuronal NCS1, supported by data mined from the GTex portal data base (https://gtexportal.org/home) **(S1 Fig**).

Using the peripheral blood cell samples that were collected over the course of the study, we obtained a non-biased assessment of changes in 484 proteins. These proteins were selected and validated by the RPPA Core of MD Anderson Cancer Center, and subsequent analyses were done using values generated by assessment of blood samples. All values obtained are in **S2 Table**. The normalized values supplied by the RPPA Core were used to construct a volcano plot [30]. Using a cut off of log_2_ (fold change from week 0 to week 12) = |0.35|, 19 proteins were increased, and 12 proteins decreased over 12 weeks of treatment (**Fig 4C**). These proteins are listed in order from the largest to the smallest change, along with an indication of increase or decrease (**Table 2**). In addition, these proteins are also sorted in order of greatest to least magnitude of fold change and split by overall increase or overall decrease for clarity **(S2 Fig).**

**Table 2:** Proteins from volcano plot which had notable increases or decreases listed in order of greatest to least significance (adjusted p-values).

PROTEIN	OVERALL	FOLD CHANGE	SIGNIFICANCE
HUWE1	**Increased**	0.34437463	1.545340633
XIAP	**Increased**	0.37434158	1.487613612
PIK3R1	**Increased**	0.44884836	0.868644852
SIRPA	*Decreased*	-0.54793455	0.847138443
XPA	**Increased**	0.49244999	0.798551469
PARG	**Increased**	0.30097693	0.795997335
PKM	*Decreased*	-0.43697609	0.731198529
GCLC	**Increased**	0.30643671	0.717533298
LCN2	**Increased**	2.13423042	0.685240753
BAK1	**Increased**	0.39620577	0.666377543
ACSS2	*Decreased*	-0.57515392	0.556066061
CD274	**Increased**	0.3421236	0.546960774
SYK	*Decreased*	-1.7181015	0.51517408
SRC	*Decreased*	-1.36674612	0.498974817
MAPK14	*Decreased*	-0.3233069	0.487154315
HSPB1	*Decreased*	-0.32123601	0.459980549
GRB2	*Decreased*	-0.38623103	0.442483739
MMP14	*Decreased*	-0.45058051	0.437104137
ACACA-B	*Decreased*	-0.30539008	0.433216936
STING1	*Decreased*	-0.67096312	0.432294581
PAK1	*Decreased*	-0.35166558	0.407032255
TFRC	**Increased**	0.37980408	0.398970288
CDH2	**Increased**	0.38266271	0.398235823
COX4I1	*Decreased*	-0.50218237	0.375319104
GYS1	*Decreased*	-0.43684964	0.31893735
SHC1	*Decreased*	-0.61168679	0.271329089
PIP4K2A	*Decreased*	-0.53142046	0.242678395

The top 15 proteins from the RPPA Core data set showing an increase were used to construct a heat map for the individual patients. Using the normalized values, the protein levels for each patient at 0 and 12 weeks were mean centered with a log_2_ transformation and a heatmap was generated based on Euclidean distance with complete linkage (**Fig 4B**). A second heatmap was generated with all proteins that had a 35% or greater change (increase or decrease) in expression from the start of treatment (week 0) to completion (week 12), based on average change across the cohort. This analysis also shows a distinct progression in protein levels **(S3 Fig).**

**Fig 5:**
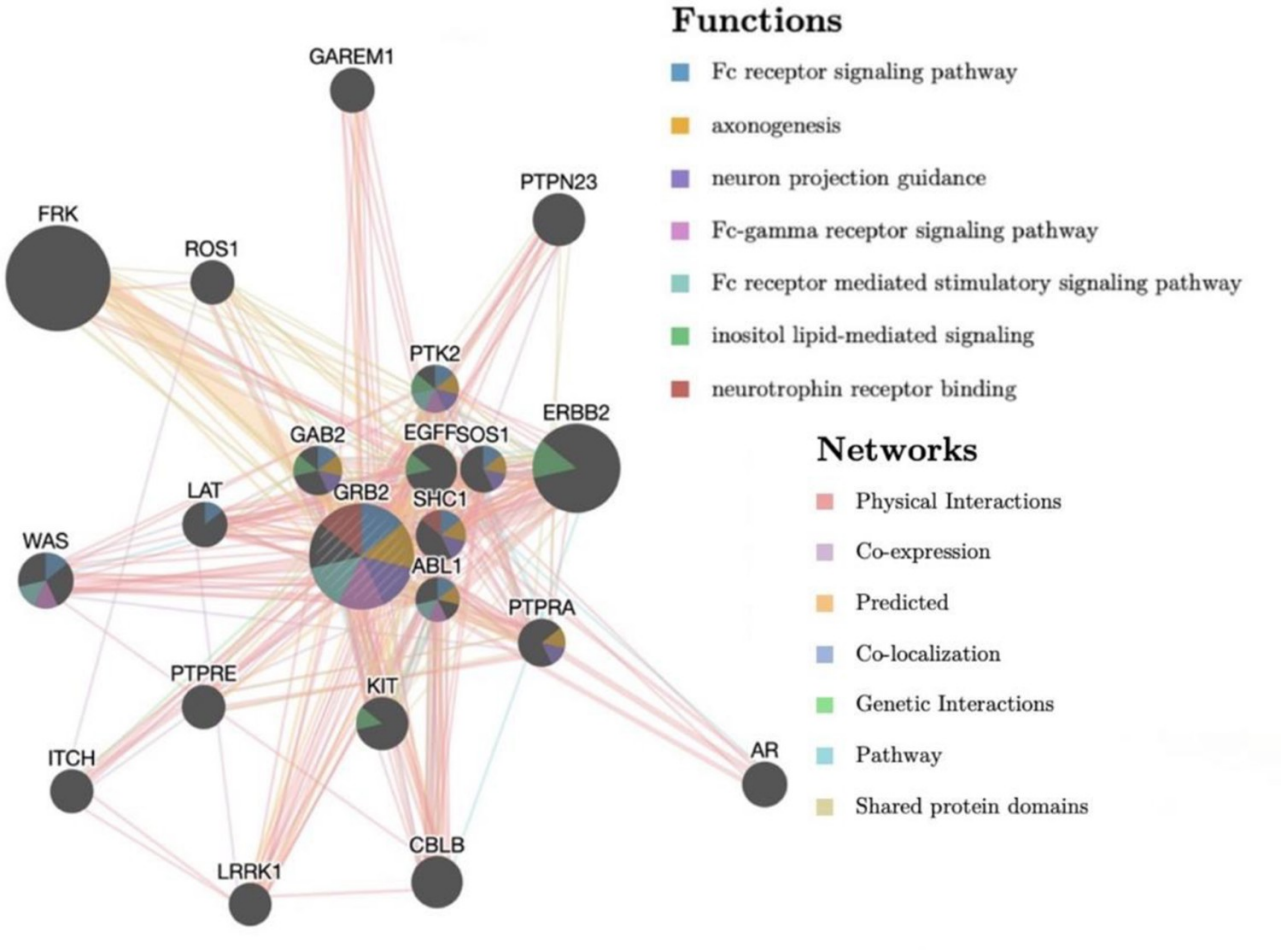
Pathway analysis using GRB2, a significantly connected and downregulated protein as the focus. GRB2 was found to have the largest number of connections with the other proteins shown in Table 2. The image of GRB2 related neighbors was generated using CPA and GeneMania. The pathway map (results with p < 0.0001) is shown along with functional pathway results which include Fc receptor signaling pathway, axoneogenesis, neuron projection guidance, inositol lipid-mediated signaling, and neutrophin receptor binding.

One of the downregulated proteins known to alter neuron viability and function, GRB2 (growth factor receptor-bound protein 2), was selected as the focus for further analysis using Consensus Pathway Analysis (CPA) and visualized using GeneMania, which organizes the pathway data using databases and literature mentions. GRB2 was selected as one of the most significant proteins in terms of relation and connection to the other affected proteins. Due to the significant connections between GRB2 and the other downregulated proteins, GRB2 was chosen as the focus for a pathway analysis. The software used, GeneMania, pulls pathway data from Reactome, IMID, Cell_Map, and HUMANCYC to determine the most significant pathways changed by taxane treatment (**Fig 5**). The closer in distance the associated proteins are in the pathway, the more of an interaction they have. GRB2 was found to have significant function within the Fc receptor signaling pathway, axoneogenesis, neuron projection guidance, Fc-gamma receptor signaling pathway, Fc receptor mediated stimulatory signaling pathway, inositol lipid-mediated signaling, and neurotrophin receptor binding. As all of these pathways are integral for both neuron viability and function, the downregulation of GRB2 is hypothesized to be related to the CIPN and CRCI seen in the patient cohort, however it is necessary to obtain more information in order to fully understand the role of GRB2 in CIPN. In addition, the inositol lipid-mediated signaling pathway is mediated by NCS1 [14], which was found to have had an overall decrease, suggesting that calcium signaling was also impacted after the course of chemotherapy.

## Discussion

Despite recent developments in breast cancer treatment, microtubule-directed drugs, including paclitaxel and docetaxel, continue to be among the most effective options for treatment of breast cancer [31]. However, taxanes are also extremely toxic for non-cancer cells, often resulting in neuronal degeneration. This neuronal side effect manifests as CIPN, a condition that is marked by numbness, loss of sensation or function in hands and feet, and muscle weakness [8]. In addition, taxane-based chemotherapy can result in CRCI, which results in attention, executive function, and memory deficits [9]. In this prospective clinical study, we aimed to examine the effect of taxanes on patients undergoing paclitaxel or docetaxel treatment through various assessments of neurotoxicity and cognitive function. **S4 Fig** presents our proposed model for the development and progression of CIPN, CRCI, and the associated side effects. We suggest that nerve degeneration in patients treated with chemotherapy is potentially linked to protein dysregulation and disrupted signaling patterns. This prospective study of 13 patients based on predetermined exclusion criteria, assessed at three time points (baseline, 6 weeks, and 12 weeks), provided insight into the manifestation of physical and cognitive side effects in patients with breast cancer through a course of taxane-based treatment. Although this study was limited by missing evaluations and sample size, the comprehensive assessments that included validated measures of neurotoxicity, direct clinical evaluations, and biochemical evaluations provide novel insight into the natural course and mechanism of neurologic toxicity in taxane treated patients [32,33].

To assess neurotoxicity, a combination of tests was used including a subjective patient-reported outcomes questionnaire (EORTC-QLQ-CIPN20) and objective neurophysiological and physical examination evaluation. The EORTC-QLQ-CIPN20 assessed everyday function and sensory related effects over the course of chemotherapy and supported the notion that chemotherapy led to neuropathy. TNS and UENS were our objective clinical evaluations and both incorporate motor and sensory tests. TNS testing has been shown to be a reliable and effective method for quantifying CIPN symptoms and monitoring severity [21]. Our data indicated that patients experienced more severe and noticeable symptoms as treatment progressed, which is consistent with the patient-reported symptoms of the EORTC-QL-CIPN20 and supports the observation that the ongoing treatment with taxanes resulted in neuropathy (**Fig 2**).

To further examine the impact of taxane on the cognitive function of breast cancer survivors, to begin to identify the affected domains, and to predict the potential risk for developing cognitive changes, we used an online cognitive assessment tool developed by CogState [34]. The CogState platform tests a variety of cognitive skills using simple tasks based on retrieval and speed. These tests demonstrated an overall decrease in learning, memory, and attention across the cohort. The decrease in cognitive function associated with taxane treatment in our patient group provides support for the notion that chemotherapy is associated with altered activity in the central nervous system, as well as in peripheral neurons.

Taxanes, including paclitaxel or docetaxel, are known to halt cell division by stabilizing microtubules [35]. But taxanes also bind to other proteins, including neuronal calcium sensor 1 (NCS1) [13], and when this binding occurs, there is an increase in intracellular calcium [13,17]. NCS1 is a high affinity, low-capacity calcium binding protein which modulates calcium signaling inside cells. NCS1 is particularly important for key functions in neurons such as dendritic arborization [18]. Previous studies have shown that exposure to taxanes triggers proteolysis of NCS1 by calpain [13,14], a ubiquitous enzyme that is activated when calcium levels are increased. When NCS1 is degraded, it becomes nonfunctional, resulting in calcium dysregulation and altered neuronal function [14–16] which is a hallmark of CIPN and CRCI. This study is the first report showing a decrease in NCS1 after taxane exposure in humans. The decrease in NCS1 levels over the course of taxane treatment in patients (**Fig 4A**) follows the findings in pre-clinical models [14–18]. From these studies, alterations in intracellular calcium signaling is a likely critical pathway leading to calcium-related dysfunction occurring during chemotherapy. Modulating the calcium signaling pathway has been a successful strategy in preclinical studies to prevent CIPN and CRCI using several drugs, lithium, ibudilast, and a PKC inhibitor [15,16].

Altered NCS1 levels have been investigated in multiple CNS disorders. NCS1 upregulation has been reported in psychiatric diseases including bipolar disorder [36] and downregulation has been observed in neuronal tissues of multiple neurodegenerative diseases such as Parkinson disease [37]. Our prior pre-clinical work has shown a strong link between NCS1 expression and neurotoxicity in mice (eg, [15,16]), and although this study is preliminary, we have aimed to address this question in human patients. Notably, NCS1 levels have been assessed as a biomarker in peripheral blood in bipolar disorder and schizophrenia [38], supporting testing NCS1 as a biomarker for CIPN and CRCI. NCS1 also has potential as a drug target for other conditions given its ability to bind signaling proteins [25,39] and small molecules [17]. Specifically, preclinical models that may show benefits by targeting NCS1 include Fragile X syndrome [40], Alzheimer’s disease [41], and Wolfram syndrome [42].

In addition to the effects on neuronal function, high expression of NCS1 in tumor tissues is associated with poor outcomes in both breast cancer and liver cancer patients [43,44]. NCS1 overexpression also is linked to enhanced tumor load in mouse models [45], likely related to a NCS1-dependent enhancement of cell motility and invasion [43,46,47]. These effects of elevated NCS1 also are consequences of altered intracellular calcium signaling.

The assessment of protein changes indicated a significant alteration in proteins associated with cancer therapy. These affected proteins encompass cell cycle regulators and tumor suppressors. Among the downregulated proteins, there is suggestion for impact on cell cycle regulation, tumor suppression, and neuronal degradation. Similarly, among the upregulated proteins, pathways associated with apoptosis are identified. In particular, the inositol-phospholipid signaling pathway (associated with the downregulated protein GRB2) is calcium regulated. In light of the NCS1 results, the connection with GRB2 to the inositol signaling pathway may elucidate the role that calcium plays in the manifestation of CIPN in taxane-treated patients **(Fig 5).** These pathways provide additional potential targets for intervention to mitigate CIPN and CRCI.

### Limitations

Although this prospective clinical study was limited in patient numbers and data collection, we were able to examine the effects of taxane treatment on patient neuronal and cognitive function longitudinally during treatment. There was an increase in neuronal dysfunction across all patients tested, but many of the values did not show large changes. This may be an indication that the tests were done too early in the development of CIPN, or that different neurological tests should have been included, or that the changes in neurological function were limited. Similarly, the decrease in cognitive function were modest. A potentially major issue is that the tests for cognitive ability are designed to detect more severe changes, such as those associated with Alzheimer’s Disease, and therefore do not have sufficient gradation to detect the brain fog associated with chemotherapy (eg, [48,49]). Additional prospective studies are needed to assess the magnitude of the changes in neuronal and cognitive function. Also, future work is required to solidify an association between protein expression levels with CIPN and CRCI phenotypes. The assessment of NCS1 levels was successful in only 3 subjects, in part because chemotherapy reduced the size of the buffy coat in the blood sample and appeared to decrease the quality of the sample. Additional precautions will be implemented for future studies. When more validated proteins have been added to the RPPA Core protein set, it will be possible to examine additional molecular pathways, especially those associated with neuronal degradation and intracellular signaling.

### Conclusion

Our results show that taxane-treated breast cancer patients are at risk for CIPN and CRCI symptoms and that these effects of taxanes become more severe and noticeable as the patients go through the full course of treatment. In the future, studies need to focus on pharmacological intervention in conjunction with the use of taxanes to mitigate the unwanted side effects of treatment. A potential intervention is related to calcium homeostasis, as our biochemical analyses indicate a downregulation of NCS1 and activation of calcium activated proteases, such as calpain. Decreasing the changes in the intracellular calcium signaling pathway would decrease the incidence of these side effects [15,16] which would have the benefit of decreasing the number of patients who chose to discontinue treatment and minimizes long-term neurologic sequelae in survivor. Ultimately, we aim to understand the role that chemotherapy plays in CIPN and CRCI on the clinical level and to formulate ways to mitigate these side effects which would provide patients with a better quality of life during and after treatment.

## Supporting information

S1 Fig Correlation of NCS1 mRNA levels in neuronal and breast samples. Each point represents an individual subject (n = 10). The line of best fit demonstrates a correlation between neuronal and breast NCS1 mRNA expression. Data from the GTEx project.
(TIF)

S2 Fig Proteins from the volcano plot which had notable increases or decreases listed in order of greatest to least magnitude of fold change and split by overall increase or overall decrease. (TIF)

S3 Fig Heatmap with proteins which had a 35% or greater change in expression from the start of treatment (week 0) to completion (week 12), n = 10. Blue indicates overall decrease in protein level, and red indicates overall increase in protein level.
(TIF)

S4 Fig A cartoon showing a mechanism for chemotherapy induced neuropathy based upon published pre-clinical results. Taxane treatment has both favorable and unwanted effects.
(TIF)

S1 Table Summary of statistics for results and tests performed. Some of the tests generate relative values that are unitless. (*For week 12, only one patient returned to give their sample for the NCS1 level measurement).
(DOCX)

S2 Table Protein values generated by the RPPA Core of MD Anderson Cancer Center. (XLSX)

S1 Raw image (PDF)
