# Supplementary figures and images for: A preliminary, prospective study of peripheral neuropathy and cognitive function in patients with breast cancer during taxane therapy
Source: PLoS One. 2022 Oct 7;17(10):e0275648. doi: 10.1371/journal.pone.0275648 (PMC9543876; doi:10.1371/journal.pone.0275648)

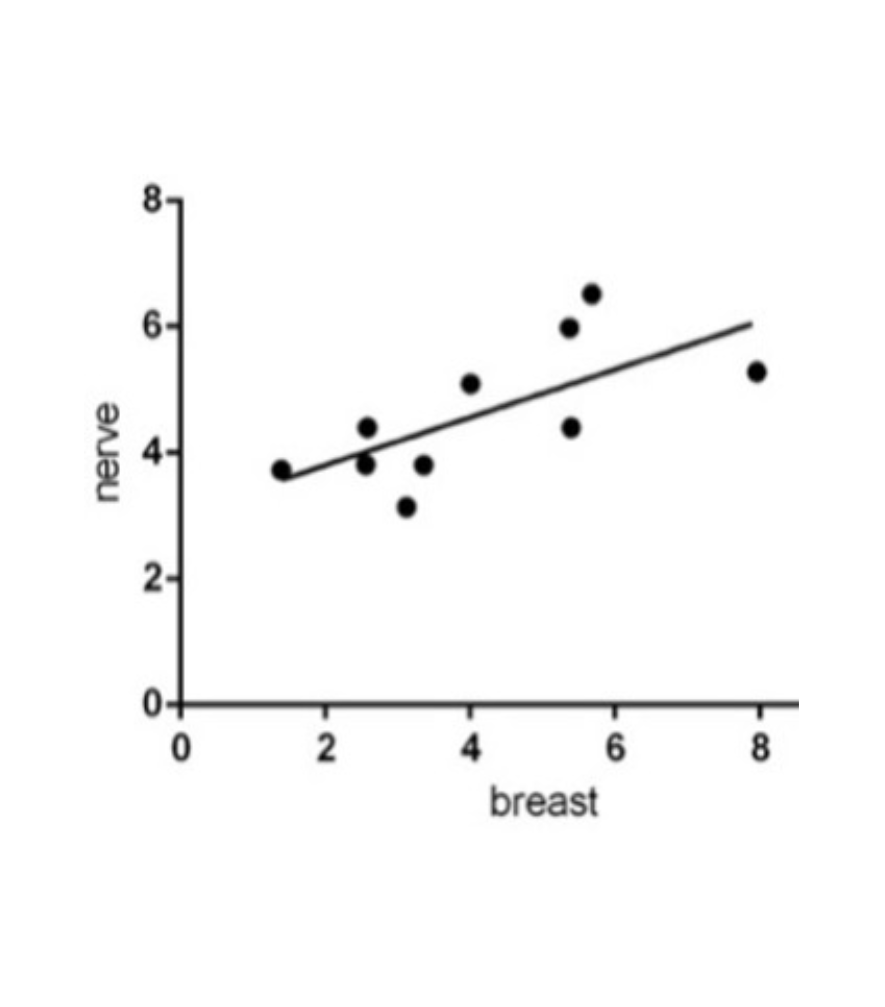

Supplement: S1 Fig — Each point represents an individual subject (n = 10). The line of best fit demonstrates a correlation between neuronal and breast NCS1 mRNA expression. Data from the GTEx project. (TIF) [file pone.0275648.s001.tif]

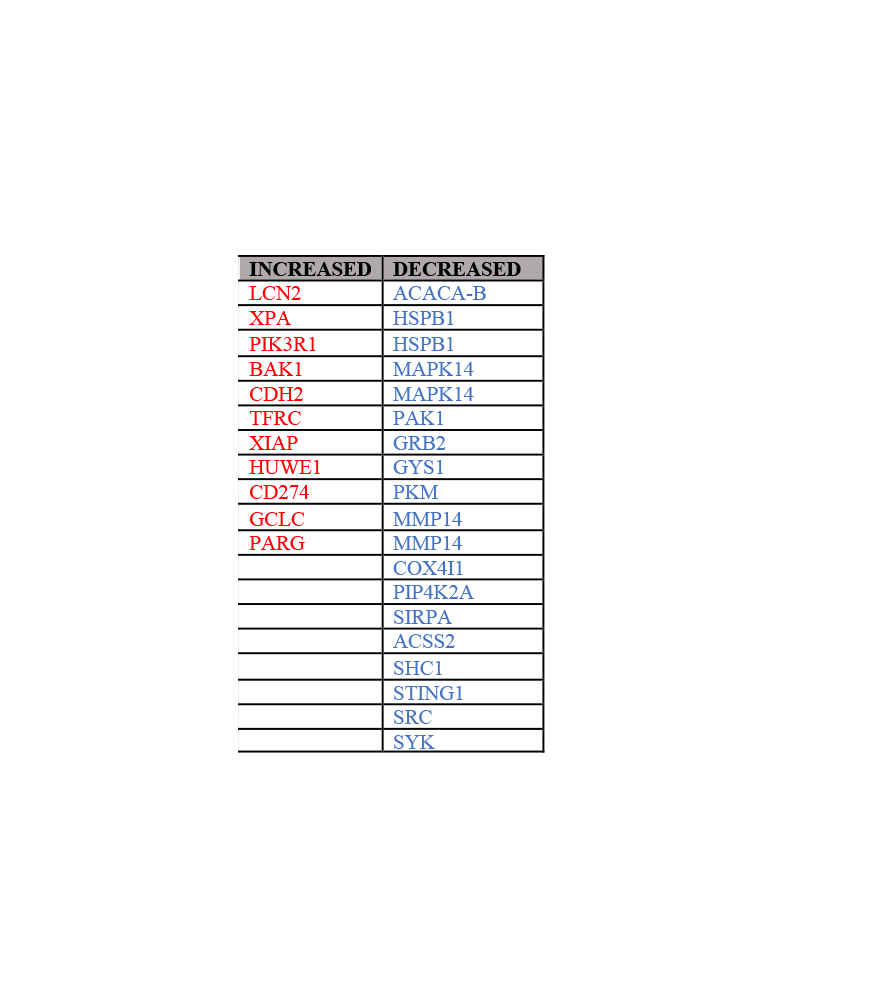

Supplement: S2 Fig — (TIF) [file pone.0275648.s002.tif]

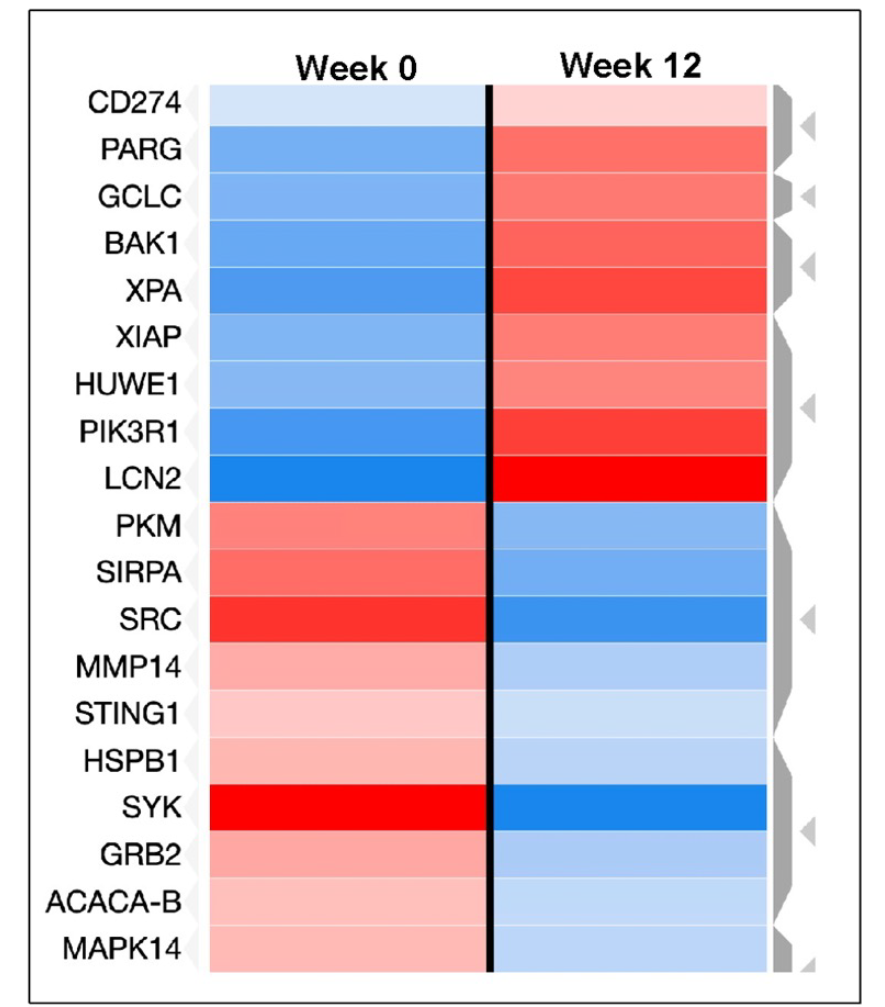

Supplement: S3 Fig — Blue indicates overall decrease in protein level, and red indicates overall increase in protein level. (TIF) [file pone.0275648.s003.tif]

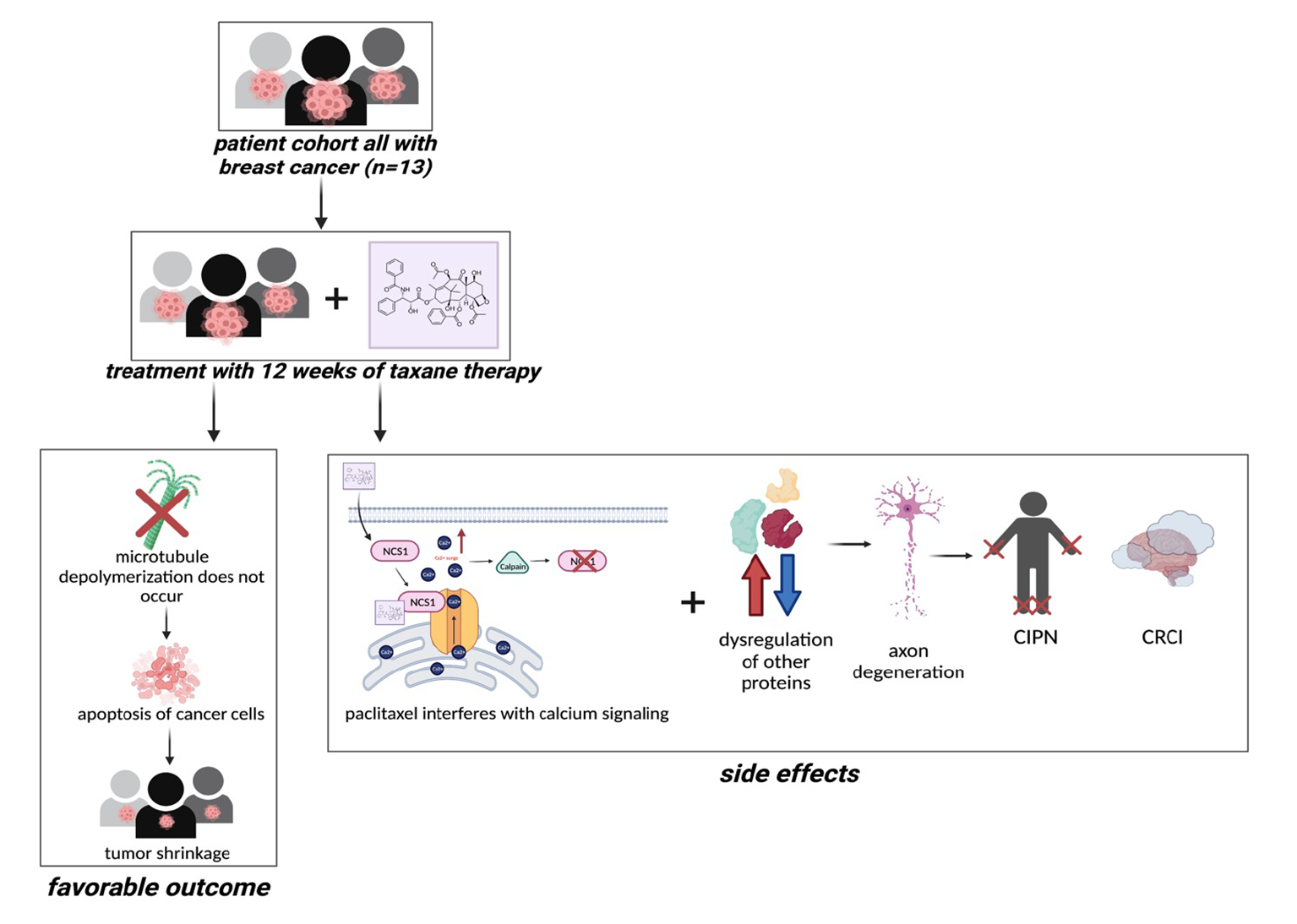

Supplement: S4 Fig — Taxane treatment has both favorable and unwanted effects. (TIF) [file pone.0275648.s004.tif]
