# Supplementary material for: A preliminary, prospective study of peripheral neuropathy and cognitive function in patients with breast cancer during taxane therapy
Source: PLoS One. 2022 Oct 7;17(10):e0275648. doi: 10.1371/journal.pone.0275648 (PMC9543876; doi:10.1371/journal.pone.0275648)
Supplement: S1 Table — Some of the tests generate relative values that are unitless. (*For week 12, only one patient returned to give their sample for the NCS1 level measurement). (DOCX) [file pone.0275648.s005.docx]

**Table S1:** Summary of statistics for results and tests performed. (*For week 12, only one patient returned to give their sample for the NCS1 level measurement).

|  | Max, Min | Mean, SD | Median | Q1, Q3 |
| --- | --- | --- | --- | --- |
|  | **Nerve Conduction Study (n=7)** | | | |
| *Sensory CV (m/s)* | 0.22, -0.25 | -0.03, 0.19 | -0.06 | -0.25, 0.15 |
| *SNAP AMP (uV)* | -0.08, -0.16 | -0.13, 0.03 | -0.15 | -0.16, -0.10 |
| *Motor Distal Latency (mS)* | 0.10, -0.16 | -0.05, 0.11 | -0.09 | -0.14, 0.06 |
| *Motor CV (m/s)* | 0.01, -0.11 | -0.06, 0.05 | -0.10 | -0.11, -0.01 |
| *CMAP AMP (uV)* | -0.04, -0.69 | -0.27, 0.30 | -0.07 | -0.54, -0.05 |
|  | **EORTC QLQ-CIPN20 (n=7)** | | | |
| *Week 0* | 36, 25 | 29, 3.3 | 28 | 27, 29 |
| *Week 6* | 41,28 | 33, 5.0 | 32 | 28, 37 |
| *Week 12* | 50, 28 | 37, 7.7 | 35 | 32, 43 |
|  | **Cogstate Assessment (n=8)** | | | |
| *Identification* | 0.01, -0.07 | -0.02, 0.02 | -0.02 | -0.04, -0.00 |
| *One Card Learning* | 0.06, -0.04 | 0.00, 0.00 | -0.01 | -0.02, 0.02 |
| *One Back Accuracy* | 0.08, -0.13 | -0.01, 0.05 | 0.00 | -0.02, 0.00 |
|  | **NCS1 Levels (n=3)** | | | |
| *Week 0 (n=3)* | 0.97, 0.53 | 0.80, 0.19 | 0.90 | 0.62, 0.95 |
| *Week 6 (n=3)* | 0.67, 0.54 | 0.61, 0.05 | 0.63 | 0.56, 0.66 |
| *Week 12 (n=1)** | 0.43 | 0.43, 0.0 | 0.43 | 0.43 |
