# Supplementary material for: A preliminary, prospective study of peripheral neuropathy and cognitive function in patients with breast cancer during taxane therapy
Source: PLoS One. 2022 Oct 7;17(10):e0275648. doi: 10.1371/journal.pone.0275648 (PMC9543876; doi:10.1371/journal.pone.0275648)
Supplement: S1 Raw image — (PDF) [file pone.0275648.s007.pdf]

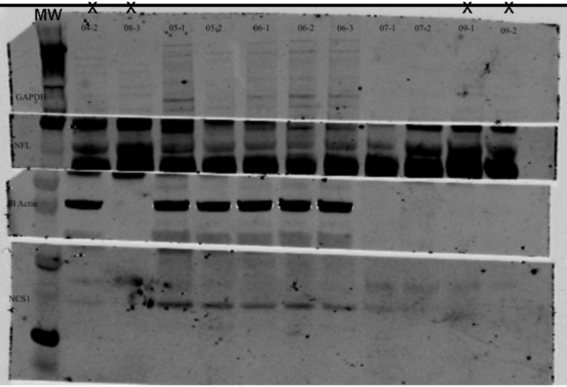

Patient number at top of each lane (Pt#-sample#)
top panel: GAPDH (not used in study)
second panel: NFL, neurofilamin light (not used in study)
third panel: B Actin
bottom panel: NCS1, neuronal calcium sensor 1

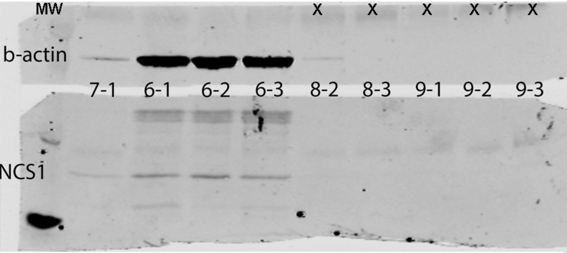

Patient number at top of each lane (Pt#-sample#)
top panel: B Actin
second panel: NCS1, neuronal calcium sensor 1
